# Basic Research in Atherosclerosis: Technologies of Personalized Medicine

**DOI:** 10.3390/jpm12030367

**Published:** 2022-02-28

**Authors:** Yuliya Ragino, Evgeniia Striukova, Elena Shakhtshneider

**Affiliations:** Institute of Internal and Preventive Medicine—Branch of Institute of Cytology and Genetics, Siberian Branch of Russian Academy of Sciences (SB RAS), 175/1 Borisa Bogatkova Str., 630089 Novosibirsk, Russia; stryukova.j@mail.ru (E.S.); 2117409@mail.ru (E.S.)

The first national conference with international participation, “Fundamental aspects of atherosclerosis: scientific research for improving the technologies of personalized medicine”, was held in Novosibirsk on 15 October 2021. The purpose of this conference was to disseminate the latest basic and clinical findings in the fields of etiology, clinical characteristics, and modern diagnostics and treatments of atherosclerosis among various relevant specialists. The conference was intended for practicing cardiologists, primary care physicians, medical geneticists, and physician–scientists. The conference included plenary sessions, specialty sessions, satellite symposia, an open competition for young scientists.

This Special Issue on “Atherosclerosis: Technologies of Personalized Medicine” includes a review and ten original studies about epidemiologic, genetic aspects of atherosclerosis, antioxidant system, biomarkers of atherosclerosis. Four of the special-issue articles, Metelskaya et al. [1], Gruzdeva et al. [2], Shramko et al. [3], Polonskaya et al. [4], focus on the various biomarkers of atherosclerosis. Metelskaya et al. [1] evaluated the feasibility of a combination of biochemical and imaging parameters for estimation of risk and severity of coronary atherosclerosis (CA), and to verify the created integrated biomarker (i-BIO) on the independent cohort. They determined that the i-BIO > 4 detected CA (GS > 0) with sensitivity of 87.9%, i-BIO ≥ 9 excluded patients without severe CA (GS < 35), specificity 79.8%. Validation of i-BIO confirmed the feasibility of i-BIO > 4 to separate patients with any CA with sensitivity 76.2%, and of i-BIO ≥ 9 to exclude atherosclerosis-free subjects with a specificity of 84.0%. Gruzdeva et al. [2] investigated the expression and secretion of adipocytokine genes in the adipose tissue (AT) of patients with coronary artery disease (CAD) and patients with aortic or mitral valve replacement. The study included 84 patients with CAD and 50 patients with aortic or mitral valve replacement. The authors revealed the pathogenetic significance of alterations in the adipokine and cytokine status of adipocytes of epicardial (EAT) and perivascular (PVAT) in patients with CAD. Shramko et al. [3] researched associations of fatty acids (FAs) with the antioxidant enzymes in the blood of men with coronary atherosclerosis and ischemic heart disease (IHD). The study included 80 patients: control group—20 men without IHD, the core group—60 men with IHD. The core group was divided into subgroups: subgroup A—with the presence of vulnerable atherosclerotic plaques, subgroup B—with the absence of vulnerable atherosclerotic plaques. The authors analyzed the levels of FAs, free radicals, superoxide dismutase (SOD), catalase (CAT), and glutathione peroxidase (GPx) in the blood and revealed that changes in the levels of antioxidant enzymes, and a disbalance of the FAs profile, probably indicate active oxidative processes in the body and may indicate the presence of atherosclerotic changes in the vessels. Polonskaya et al. [4] investigated the relationship of matrix metalloproteinases with calcification of the coronary arteries. The study included 78 people with coronary heart disease (CHD) and 36 without CHD. Blood and samples of coronary arteries obtained as a result of endarterectomy were examined. Serum levels of metalloproteinases (MMP) MMP-1, MMP-2, MMP-3, MMP-7, MMP-9, MMP-10, MMP-12, and MMP-13 were determined by multiplex analysis. In blood vessel samples, MMP-1, MMP-3, MMP-7, and MMP-9 were determined by enzyme immunoassay; MMP-9 expression was evaluated by immunohistochemistry. The results obtained indicate the participation of some MMPs, and especially MMP-9, in the calcification processes. The study can serve as a basis for the further study of the possibility of using MMP-1, MMP-7 and MMP-12 as potential biomarkers of CHD.

Afanasieva et al. [5] focused on the relationship between Lp(a), immune blood cells and major adverse cardiovascular events (MACE) in patients with the early manifestation of coronary heart disease (CHD). The study included 200 patients with chronic CHD, manifested up to the age of 55 in men and 60 in women. An increased Lp(a) concentration [hyperLp(a)] was shown to predict cardiovascular events in patients with premature CHD with long-term follow-up. The combination of an increased monocyte count and hyperLp(a) significantly increased the proportion of patients with early CHD with subsequent development of MACE. The odds of cardiovascular events in patients with early CHD manifestation were highest in patients with an elevated lymphocyte-to-monocyte ratio and an elevated Lp(a) level. A higher neutrophil blood count and an elevated neutrophil-to-lymphocyte ratio determined the faster development of MACE in patients with a high Lp(a) concentration. The authors suggested that the high atherothrombogenicity of Lp(a) is associated with the “inflammatory” component and the innate immune cells’ involvement in this process. The easily calculated immunological ratios of blood cells and Lp(a) concentrations can be considered simple predictors of future cardiovascular events.

Shapkina et al. [6] investigated the determinants of the 13-year risk of incident atrial fibrillation (AF) in a Russian population cohort of middle and elderly age. A random population sample (*n* = 9360, age 45–69 years) was examined at baseline in 2003–2005 and reexamined in 2006–2008 and 2015–2017 in Novosibirsk (the HAPIEE study). Incident AF was being registered during the average follow-up of 13 years. The final analysis included 3871 participants free from baseline AF and cardiovascular disease (CVD) who participated in all three data collections. In a multivariable-adjusted Cox regression model, the 13-year risk of AF was positively associated with the male sex, age, body mass index (BMI), systolic blood pressure (SBP), and it was negatively associated with total cholesterol (TC). In women, the risk of AF was more strongly associated with hypertension (HT) and was also negatively related to total cholesterol (TC) level. No independent association was found with mean alcohol intake per drinking occasion.

Malyutina et al. [7] evaluated the relationship between ‘epigenetic age’ (EA) derived from DNA methylation (DNAm) and myocardial infarction (MI)/acute coronary syndrome (ACS). A random population sample was examined in 2003/2005 (*n* = 9360, 45–69, the HAPIEE project) and followed up for 15 years. From this cohort, incident MI/ACS (cases, *n* = 129) and age- and sex-stratified controls (*n* = 177) were selected for a nested case-control study. Baseline EA (Horvath’s, Hannum’s, PhenoAge, Skin and Blood) and the differences between EA and chronological age (CA) were calculated (ΔAHr, ΔAHn, ΔAPh, ΔASB). EAs by Horvath’s, Hannum’s and Skin and Blood were close to CA (median absolute difference, MAD, of 1.08, −1.91 and −2.03 years); PhenoAge had MAD of −9.29 years vs. CA. The adjusted odds ratios (ORs) of MI/ACS per 1–year increments of ΔAHr, ΔAHn, ΔASB and ΔAPh were 1.01 (95% CI 0.95–1.07), 1.01 (95% CI 0.95–1.08), 1.02 (95% CI 0.97–1.06) and 1.01 (0.93–1.09), respectively. When classified into tertiles, only the highest tertile of ΔAPh showed a suggestion of increased risk of MI/ACS with OR 2.09 (1.11–3.94) independent of age and 1.84 (0.99–3.52) in the age- and sex-adjusted model. The authors concluded that in a prospective population-based cohort there were no strong associations between accelerated epigenetic age markers and risk of MI/ACS.

Korneva et al. [8] analyzed the contribution of “cum LDL-C for all life” and the index “cum LDL-C/age” to the development of coronary heart disease (CHD), myocardial infarction (MI), and a combined endpoint: MI, stroke, unstable angina in FH patients. The study included 188 patients (mean age 49.2 years, males 45.7%) with FH were examined (Dutch Lipid Clinic Criteria). The authors had evaluated cumulative LDL-C and index “cum DL-C/age” along with other classical risk factors. Cum LDL-C was calculated as LDL-Cmax × (age at initiating of hypolipidemic therapy) + LDL-C at inclusion age at initiation/correction therapy). Cumulative LDL-C and “cum LDL-C/age” were calculated as the ratio cum LDL-C to age. The follow-up period was 5.4 (from 3 to 10) years. The index “cum LDL-C/age” was higher in patients with CHD 58.7 ± 10.4 mmol/L/years vs. 40.1 ± 11.7 mmol/L/years in patients without CHD. According to their data based on the results of the logistic regression analysis in patients with FH, cumulative LDL-C and the cumulative index “cum LDL–C/age” played a strong predictive role in the development of CHD in FH patients; it was greater than the role of TC and LDL-C concentrations. The authors suggest that cumulative LDL-C level plays an important role in the development of CHD in FH patients.

Shakhtshneider et al. [9] focused on the genetic variants potentially involved in familial hypercholesterolemia in 43 genes associated with lipid metabolism disorders. Targeted high-throughput sequencing of lipid metabolism genes was performed (80 subjects with a familial-hypercholesterolemia phenotype). For patients without functionally significant substitutions in the above genes, multiplex ligation-dependent probe amplification was conducted to determine bigger mutations (deletions and/or duplications) in the *LDLR* promoter and exons. A clinically significant variant in some genes associated with familial hypercholesterolemia was identified in 47.5% of the subjects. Clinically significant variants in the *LDLR* gene were identified in 19 probands (73.1% of all variants identified in probands); in three probands (11.5%), pathogenic variants were found in the *APOB* gene; and in four probands (15.4%), rare, clinically significant variants were identified in genes *LPL*, *SREBF1*, *APOC3*, and *ABCG5*. In 12 (85.7%) of 14 children of the probands, clinically significant variants were detectable in genes associated with familial hypercholesterolemia. The use of clinical criteria, targeted sequencing, and multiplex ligation-dependent probe amplification makes it possible to identify carriers of rare clinically significant variants in a wide range of lipid metabolism genes and to investigate their influence on phenotypic manifestations of familial hypercholesterolemia.

Khan et al. [10] evaluated how ASA non-sensitivity can be diagnosed using Plateletworks^®^, a point-of-care platelet function test. Patients prescribed 81 mg of ASA were recruited in a series of two successive phases—a discovery phase and a validation phase. In the discovery phase, a total of 60 patients were recruited to establish a cut-off point (COP) for ASA non-sensitivity using Plateletworks^®^. Each sample was simultaneously cross-referenced with a light transmission aggregometer (LTA). Their findings demonstrated that >52% maximal platelet aggregation using Plateletworks^®^ had a sensitivity, specificity, and likelihood ratio of 80%, 70%, and 2.67, respectively, in predicting ASA non-sensitivity. This COP was validated in a secondary cohort of 40 patients prescribed 81 mg of ASA using Plateletworks^®^ and LTA. The data demonstrated that established COP had a 91% sensitivity and 69% specificity in identifying ASA non-sensitivity using Plateletworks^®^. The authors suggested that Plateletworks^®^ is a point-of-care platelet function test that can appropriately diagnose ASA non-sensitive patients with a sensitivity exceeding 80%.

The articles in this special issue cover interesting topics in lipidology that are also related to cardiology, internal medicine, genetics, and epidemiology. The presented data expand our knowledge about atherosclerosis.
